# Minority joy, minority growth, and minority peace: Transgender and gender diverse people’s varied positive lived experiences

**DOI:** 10.1080/26895269.2024.2445094

**Published:** 2024-12-27

**Authors:** Matilda Wurm, T. Lundberg, T. Mejias Nihlén, A. Malmquist

**Affiliations:** a School of Behavioural, Social and Legal Sciences, Örebro University, Örebro, Sweden; b Department of Psychology, Lund University, Lund, Sweden; c Department of Behavioral Sciences and Learning, Linköping University, Linköping, Sweden

**Keywords:** Gender affirming care, quality of life, reflexive thematic analysis, resilience, trans joy

## Abstract

**Background:**

Compared to negative experiences and ill health, little attention has been paid to positive experiences connected to being transgender and gender diverse (TGD).

**Aim:**

The aim of the current study was to explore TGD people’s positive experiences in depth.

**Methods:**

In total, 33 TGD people were interviewed. Transcripts were analyzed using reflexive thematic analysis.

**Results:**

Results show that participants had various positive experiences related to their gender identity. While some of these experiences can be conceptualized as “minority joy,” capturing experiences evoking happy emotions, others are better understood as “minority growth”—a personal development and sense of authenticity—and “minority peace”—a sense of comfortability and calmness. The emotional valence of experiences was further explored to better understand the themes.

**Discussion:**

In line with earlier research, some of the positive experiences could be seen as buffers between stressors and ill health and some were clearly connected to experiencing stressors. However, some experiences were freestanding from stressors and positive in their own right. The results of this study give an alternative to the prevailing view of TGD experiences necessarily connected to suffering. The results are relevant for professionals meeting TGD people, since a focus on strengths and positive aspects could be an important, but previously under focused tool to improve general health and quality of life. It may give hope to TGD people themselves, who are often presented with the trope of the suffering TGD person. Not the least, results could inform general society and policy makers.

In recent decades, health issues in gender and sexual minorities have received increased attention, since these groups show higher levels of physical and mental ill health compared to the heterosexual cisgender majority (Frost & Meyer, 2023). Transgender and gender diverse (TGD) individuals show particularly high rates of ill health (e.g. Fredriksen-Goldsen et al., 2014; Valentine & Shipherd, 2018; Williams et al., 2021). The concept of minority stress has been used to explain these health disparities (Linander et al., 2024; Meyer, 2003). While initially developed with a focus on sexual minorities (Meyer, 2003), further developments of minority stress theory specifically address stressors among TGD people (Testa et al., 2015), highlighting that some are similar to those experienced by sexual minorities, while others are unique or qualitatively different.

Understanding ill health among TGD people is crucial. However, focusing solely on stressors, health problems, and negative experiences has created what Shuster and Westbrook (2024, p. 2) have called the “trope of the transgender person in misery.” This is problematic, since it overlooks the varied experiences of TGD individuals, and disregards positive experiences of being TGD. Expectations of negative outcomes and experiences may influence both professionals, TGD people, and their close ones negatively. Holloway (2023) recently formulated a call to practitioners, researchers, and educators stressing the importance of shifting trans narratives from viewing TGD people as “damaged” to focusing on strengths. This mirrors the observation that minority stress and good general health often coexist. For example, even though negative experiences were also described, 51% of TGD respondents in Sweden reported good general health when filling in a self-report questionnaire (Zeluf et al., 2016).

Some attention has already been paid to protective factors, such as healthy coping strategies and community support (Frost & Meyer, 2012; Meyer, 2003). These explain how people can do well, despite experiencing minority stress. Related to this, The Swedish National Board for Social Affairs and Health (2024) highlights the importance of understanding ill health and well-being as two separate constructs that may be present simultaneously. However, when positive factors are only understood as resilience or protective factors, the trope of the trans person in misery prevails. In clinical practice, therefore, there is a risk that positive aspects may not be focused, and tools aiding recovery may be underutilized.

In contrast to this understanding, good mental and physical health and high quality of life, as experienced by many TGD people, may be caused by a variety of positive aspects, beyond an ability to cope with stressors. In addition to the concept “minority stress” we have suggested the concept “minority joy,” aiming at broadly capturing positive experiences that are related to the minority experience (Wurm et al., 2022). With the purpose of building empirical evidence on positive aspects of belonging to a sexual and/or gender minority, the aim of this study was to inductively explore what positive experiences TGD people have, *because* they are TGD, that is, experiences they would not have had if they were cisgender. In this study we use the term *transgender and gender diverse* (TGD). The term includes a variety of experiences and identities but does not imply that each individual necessarily incorporates diverse identities.

### Background

While most TGD psychology and sociology research has focused on stressors and ill health, there is a growing number of studies focusing on protective and positive factors in TGD populations. These studies generally aim at exploring one or more aspects of protective or positive experiences departing from concepts such as community connectedness, and gender euphoria (Frost & Meyer, 2012; Pflum et al., 2015; Sherman et al., 2020). Community is crucial for promoting resilience and buffering against ill health, fulfilling the basic human need for belongingness (Frost & Meyer, 2012). The concept of “gender euphoria,” has been explored as the opposite of “gender dysphoria” regarding the emotional valence (Beischel et al., 2022). Gender euphoria refers to the relief and joy when discomfort due to gender dysphoria disappears, for example during gender-affirming treatment (Bradford et al., 2021). It also includes joy due to the alignment between one’s gender identity and gender-specific attributes experienced physically (e.g. appearance), psychologically (e.g. feelings), or socially (e.g. being affirmed as one’s gender identity) (Ashley & Ells, 2018; Austin et al., 2022; Beischel et al., 2022). Gender dysphoria and gender euphoria may be experienced separately or simultaneously and may be intertwined as diminished gender dysphoria in itself may be experienced positively (Beischel et al., 2022). Both community connectedness and gender euphoria have been understood as connected to resilience, that is, the ability to strive despite experiencing minority stress (Grant et al., 2024; Wilson et al., 2024).

While the term “trans(gender) joy” is used by some scholars (Flynn et al., 2024; Patel, 2024; Shuster & Westbrook, 2024), it has not been clearly defined, but has been used with the attempt of exploring general positive experiences and expressions of TGD people or to problematize the general focus on negative experiences (Flynn et al., 2024; Patel, 2024; Shuster & Westbrook, 2024). Nevertheless, few studies empirically explore positive aspects of TGD identity broadly. One exception is Riggle et al. (2011) where 97% of TGD participants answering a questionnaire reported that they experienced their trans identity as somewhat or very positive. Positive experiences included being a congruent self, a strengthening of relationships when being in an affirming environment, development of an inner strength they had not accessed before transitioning, and increased empathic ability, which led to increased involvement in issues that were important to them. Being part of the LGBTQI+ community was also an important source of joy and support. Another study, by Flynn et al. (2024), analyzed responses from 94 TGD participants on an open-ended survey question about positive experiences related to their identity. Results highlighted experiences of social support, romantic and sexual relationships, community connectedness, exploring and disclosing identity, and gender expression and confirmation. Finally, Shuster and Westbrook (2024) concluded interviews on transgender experiences with a question on positive experiences. Results showed that participants enjoyed being part of a marginalized group, and that being TGD raised quality of life and facilitated meaningful connections to others.

While these previous studies explore positive aspects of TGD identities broadly, the data consists of shorter written answers (Flynn et al., 2024; Riggle et al., 2011) or the concluding question of an interview not otherwise focusing on positive aspects (Shuster & Westbrook, 2024). Conducting interviews specifically focused on positive experiences would give TGD people more space to reflect and could provide valuable insights into the field.

## Methods

### Design

With the aim of exploring positive experiences, semi-structured interviews were conducted and analyzed with critical realist reflexive thematic analysis (Braun & Clarke, 2006, 2019).

The current study complies with the 1964 Helsinki Declaration and was approved by the Ethical Review Authority in Sweden (2022-04823-01). The study is part of a project funded by the Swedish Research Council for Health, Working life and Welfare (FORTE, 2021-01944).

### Positionality

This study was conducted by the research group Queer Psychology Sweden (QueerPsy), which mainly focuses on research on LGBTQ+ health and well-being.

The authors of this specific paper have combined expertise in health psychology, qualitative methodology, social psychology, sexology, and clinical psychology. We are part of different LGBTQ+ communities, and our research is informed by these lived experiences. In addition, we maintain an ongoing dialogue with LGBTQ+ organizations across the different stages of our projects.

### Procedure

Participants were recruited using convenience sampling *via* social media ads targeting LGBTQI+ and TGD groups in January 2022. The inclusion criteria were self-identifying as TGD or having TGD experience. The ad provided brief study details, and 50 interested individuals e-mailed the researchers. After receiving more information, 11 either declined participation due to lack of time or did not respond to emails. With the available resources and an ambition to account for variation, we aimed for 30 interviews with a roughly equal number of trans men, trans women, and nonbinary identities. Because of the high interest, we conducted 33 interviews, which is in line with recommendations for reflexive thematic analysis (Clarke & Braun, 2017). Due to lack of recourses and an uneven distribution of TGD identities, the remaining individuals (identifying as trans men, or nonbinary) were thanked for their interest and informed that we could not interview everyone.

### Materials

After informed consent was received, semi-structured interviews were conducted *via* Zoom, which allowed participation across Sweden. Interviews lasted 30–69 min (*M* = 48) and were conducted between February and December 2022 by one of the researchers and two master students at the end of their clinical psychology training.

During the interviews, an interview guide was used including questions on demographic information and a prompt to recall positive experiences connected to being TGD (“What advantages do you experience that you have because you are [insert term used by participant, e.g. nonbinary] and what positive experiences have you had that you would not have had if you were cis?”). The guide also included follow-up questions to explore these experiences further. Participants were first asked to brainstorm and write down positive experiences related to their TGD identity in the available chat function. This was done to ensure that participants freely recalled all kinds of positive experiences, without being influenced by interviewers’ previous understanding of the topic area. The interviewer then asked the participants questions about demographic information after which participants were asked to describe and reflect on each written point.

### Participants

Among the 33 participants, 14 identified as men/trans men, 8 as women/trans women, and 11 had a variation of nonbinary identities (such as nonbinary, genderfluid, agender, non-categorized gender). All but one of the nonbinary participants were assigned female at birth. Some used multiple labels for their gender identity. Ages ranged from 20 to 64 (*M* = 34). Roughly half of the trans men and nonbinary participants, but only two of the trans women lived in larger cities. Three participants lived abroad (in Europe, Asia, and North America). Three were born in other countries in Europe, the remaining thirty were born in Sweden. Most trans men and nonbinary participants, but only half of the trans women had a university education or were studying at the time of the interview. Roughly half of the participants were in (a) romantic relationship(s). Regarding sexual identity, two described themselves as asexual, six as queer, five as bi/pan, eight as homosexual/gay, two as heterosexual, two simultaneously used gay or homosexual *and* queer, one asexual and panromantic, and some did not describe their sexual identity or used no labels. Additionally, three described being polyamorous. When asked about belonging to other minorities three reported belonging to ethnic minorities, three mentioned not being neurotypical, and three mentioned a chronic physical illness.

### Data analysis

Audio files were transcribed verbatim and pseudonymised. Two psychology students conducted exploratory preliminary analyses for two separate master’s theses based on the first 19 interviews (Ekesbo, 2023; Filis, 2022). Both coded their data sets inductively using thematic analyses following the six steps described in Braun and Clarke (2006). The interpretative leap from codes to broader themes was supervised by the last author.

For the work on this paper, the previous thematizations were synthesized into a new thematic structure. Thereafter, the last 14 interviews were read carefully, and coded deductively guided by the new developed themes using reflexive thematic analysis (Braun & Clarke, 2006, 2019; Clarke & Braun, 2017). With the addition of these transcripts, the thematic structure was revised again by the last author, and discussed between the researchers, until the final thematic structure was reached. The discussion included careful reading by the first and second author, and shared reflections on the meaning bearing structures of the themes and how to understand them. During these analytic discussions, subthemes were amended to better fit this structure and the diverse interpretations of the data by all researchers. This process enabled us to revisit the data reflexively and to get a better understanding of the meaning and emotional valence of the themes, in line with the suggestions of Braun and Clarke (2019). For example, following this reflexive process, the interpretation of the meaning of community illustrated two separate emotional states, which were then integrated into two different main themes. Finally, the results section was written up, and illuminative quotes were selected to illustrate the findings.

## Results

The findings are divided into three major themes (see Table 1), each covering what we interpret as fundamental positive aspects of being TGD. Noticeably, the experiences our participants talked about contained different emotional valences, which led us to expand on our preliminary main concept of minority joy. The first theme, *Minority joy,* displays life experiences that have been interpreted as happy and joyful, or as felt advantages of being TGD that have resulted in feelings with comparable valences. In the second theme, *Minority growth,* emphasis is on experiences described as a sense of growing as a human being. Emotionally, this is interpreted as being related to contentment, pride, and a feeling of being grounded and living authentically. The third theme, *Minority peace,* illustrates feelings of relaxation and calmness, both in contact with affirming others, and in a stable self. Each theme includes three or four sub-themes.

**Table 1.** Overview of themes constructed using reflexive thematic analysis.

Main themes	Sub-themes
Minority joy	Enjoying community
	Gender euphoria
	Gender freedom and playfulness
	Gender privilege
Minority growth	Feelings of being authentic
	Personal development
	Contributing to a better society
Minority peace	Resting from mental ill health
	Relaxing in safe environments
	Personal stability

### Minority joy

The theme minority joy was articulated in the analysis to cover a variety of pleasurable and happy life events and sensations related to being TGD. The subtheme *Enjoying community* was constructed to focus on the positive social aspects of belonging to a TGD community. The subtheme *Gender euphoria* illustrates participants’ accounts of the euphoric feeling from bodily changes, gendered attributes and/or social affirmation. The subtheme *Gender freedom and playfulness* captures participants’ descriptions of joy and fun from experimenting with gender expressions, and feeling a freedom from gendered boundaries. The subtheme *Gender privilege* covers privileges gained either from the assigned gender or from the lived gender.

#### Enjoying community

When brainstorming positive aspects of being TGD, many participants first mentioned being connected to a TGD community. The community was depicted as a space where people are “very open and have no prejudice” (Maria, woman, 40), where you can feel “connection to others […] you are somehow never alone” (Nils, man, 25). Michele described this as an “amazing experience” and continued:

[It is] the greatest benefit of knowing there’s a community out there that says, "I am valid, and there are people who feel the same way I do, and there are also people who don’t feel like I do, and we still belong together”. (Michele, agender, 22)

Michele described the feeling of belongingness when being among others who share having TGD experiences as “amazing,” and many others agreed. As another participant stressed,” belonging to a minority creates a very strong ‘in-group’” (Mika, non-categorized gender, 32). Some emphasized that TGD and/or wider LGBTQI+ communities were loving and fun, like Eli (non-categorized gender, 41) who felt that “LGBT people are trained in that, to sort of spread messages of love, and also, yeah, to produce love in a way.” Belonging to a community where “producing” and “spreading” love is at the center had been very enjoyable for Eli, who also highlighted the joy of “festivals, friends and parties.” The community also served as a source of inspiration when meeting “those who pave the way, who dare” (Hans, man, 28) and being able to “explore others’ stories” (Lejla, woman, 24) or “create a space for trans people of the future, and honor those who were here before us” (Michele, agender, 22). Nils emphasized the values of a cross-generational community:

The exchange of experiences that you can get from, you know, talking to people across generational boundaries, […]. Because there are people in their sixties who have just come out, and then there are people in their twenties who came out when they were, like, small children. So, there’s a lot of different experiences there, and, you know, different knowledge that can be shared. (Nils, man, 25)

Nils highlighted the unique richness of experiences of a cross-generational TGD community, which, to him, was an important positive aspect.

### Gender euphoria

Many participants described strong euphoric feelings, especially connected to the early periods of transitioning, such as their first experiences of being affirmed in their gender. Some described euphoric feelings about their bodies when results of gender affirming healthcare became visible. Liam was one of them:

Sometimes I just find myself standing there, getting a bit absorbed by the changes, thinking, “Wow, I mean, this is, it’s like, it’s all falling into place somehow. (Liam, man, 51)

For Liam and many others who had undergone hormonal treatment, seeing their reflection in the mirror brought strong positive emotions.

Besides bodily changes, several participants described strong euphoric feelings when they began wearing gendered clothes or engaging in gendered activities, like wearing make-up, or trying out a new dress. This led to an “almost indescribable strong feeling […] I even cried tears of happiness” (Lejla, woman, 24). Linnea described her feeling when she began wearing “women’s clothing”:

I had a cape, I guess you could call it, and it was so wonderful to have one’s clothes blowing in the wind. And after that, trying skirts and things like that. Also, […] I couldn’t stop giggling or just having a big smile on my face from wearing them and feeling the movement of things. (Linnea, woman/nonbinary, 30)

Because of the strong affirmative feelings gendered attributes may bring, some participants described how they often put on a favorite piece of clothing when they felt low, as “It will lift my spirits, wearing [my first dress]” (Lejla, woman, 24).

A euphoric feeling was also commonly described when participants’ gender identity was affirmed by others. William (man, 20) experienced this when he “was called ‘son’ by my dad, ‘brother’ by my brother” and “the first time I heard my correct pronouns from friends.” While some participants, like William, experienced positive feelings when being correctly gendered by people who knew them, others felt particularly strong positive emotions when strangers correctly assumed their gender. Edvin described:

[There was] someone speaking English who called me "sir," and it was just like, "oh." I hadn’t expected that. It really took me by surprise. […] I don’t really know how to describe the feeling, […] it’s like both ecstasy and calm at the same time. (Edvin, man, 28)

As he had not expected to be correctly addressed as “sir,” Edvin experienced a strong emotional reaction of both intense and calm feelings at once. Being correctly gendered, especially when not used to it, was often described as a very pleasurable feeling, as being weightless or without friction. For some participants, particularly strong emotions were felt when their gender was affirmed in specific situations. Nils (man, 25), who identified as gay, felt a “nice feeling” when cisgender gay men showed attraction toward him, because he “knew they were attracted to men, and masculine men, and that I, then, am included in that.”

Some participants highlighted the positive feelings of being included in gendered communities. As Pontus (man, 52) said, “some guys take the lead, and then a few follow, and in the end it’s like ‘Pontus is one of us’.” Likewise, Josefin (woman, 64) said it felt great to be welcomed into the female community and to be seen as “one of the girls.” Thus, a transition may give access to new communities, and these may also be found among cisgender people.

### Gender freedom and playfulness

Many nonbinary participants emphasized how an identity beyond the binary norm allowed them to be playful and experiment with gender expressions and behaviors. They felt a social freedom where they could form their identity without having to care about gender stereotypes, as Sasha (nonbinary, 32) explained “It’s freedom […] no one expects, and I don’t do either, that I would be like anyone else.” Likewise, Mika, said:

It can also be a euphoric feeling, I mean, there can be a lot of joy in it, like, “I don’t have to do that,” “I don’t have to be part of that,” and “what I have is so much freer and so much kinder, and so much larger, in a way”. (Mika, non-categorized gender, 32)

Mika had realized that a non-categorized gender identity allowed them to freely decide who they wanted to be and how to behave. This freedom also affected Mika’s romantic and sexual relationships, as “there is so much space to stage things and formulate things”, once you break free from gendered norms, and find space to “experiment and play.”

While the joy of playing with gender was most commonly described by nonbinary participants, some with binary identities also enjoyed playing with gender expressions and norms. Some transgender men felt relaxed and joyful when (re)claiming femininely coded activities, such as knitting or wearing make-up, once they felt more settled in their male gender. David had transitioned socially but was most often read as a woman in public. He described how he would often “pick the best from two sides”, and exemplified with public restrooms:

It’s a lot cleaner in the women’s restrooms [laughs], but the lines are shorter to the men’s restrooms, so, [laughs] if I want a nice restroom, I’ll go to the wom-, and otherwise, if there’s a short line, I can go to the men’s, and if people just think [that it’s inappropriate], I go “Oh, well, my name is David.” (David, man, 45)

In contrast to how many TGD people experience gender separated restrooms as a major stressor (Lewkowitz & Gilliland, 2025), David took advantage of the ability to use any restroom.

### Gender privilege

Participants described privileges they experienced from the gender role they were socialized in or the gender they were living in. Some also highlighted specific gender privileges stemming from being TGD. Josefin spoke about male privilege:

I lost a lot of male privileges, but I still carry a bit with me from my upbringing. Boys are taught to take up space and be heard and seen, and you can also see that, you know, trans women are often more forward in many contexts compared to trans men, although there are exceptions, of course. (Josefin, woman, 64)

While Josefin felt that she and other trans women benefited from male privilege from being socialized as boys, Jens (man, 36) had gained male privilege later in life and was astonished when he realized how others treated him with respect, as “Suddenly, I noticed that most men […] believed what I was saying! Without even checking if what I said was true.” Thus, the advantages of male privilege were described as beneficial for both trans men and trans women.

Some, particularly among the nonbinary participants, described feeling privileged because they were not limited to one stereotypic gender role. Yoshi explained this:

It feels like possibilities open up because I’m nonbinary and gender is irrelevant to me personally. […] Instead of just having half a buffet, I have the whole buffet, even more. […] Yeah, it feels like having endless possibilities, and that’s very liberating, it’s really nice. (Yoshi, nonbinary, 26)

Yoshi described the nonbinary identity as giving them access to” the full buffet”, rather than” a half buffet.” Similarly, several other participants emphasized the specific privilege of not being limited by gender stereotypes.

## Minority growth

The theme minority growth highlights participants’ experiences of growing, maturing, and developing new skills due to their TGD experiences. While many recounted difficult experiences, they here described appreciation of the personal insights and development they achieved. The theme has three subthemes. The subtheme *Being authentic* emphasizes the deeply meaningful experience of being your true self. *Personal development* focuses on maturing through challenges and gaining unique insights related to being TGD, which participants felt led to open-mindedness and norm-critical perspectives. The subtheme *Contributing to a better society,* focuses on the participants’ experiences of using insights to improve conditions for others.

### Feelings of being authentic

Most participants described a deeply meaningful experience of authenticity connected to being TGD. This could lead to” incredible happiness […] because I found myself and there were words to describe me and my reality” (Hans, man, 28). Being true to oneself was by some described as the most fundamental aspect of being TGD, more important than external acceptance or validation. Many valued transitioning as a life changing process that required deep reflection. Anders explained:

It might not be common for people to be forced to sit down at some point and really think through "Who am I?", you know, "What am I doing?" in such a fundamental way. This is something you have to go through if you’re deciding first whether to undergo the entire gender changing process and then actually go through it. […] I feel much more certain about the fundamental things I’ve needed to think through. (Anders, man, 42)

For Anders, the decision to transition had led to a deeper understanding of himself. Likewise, William stressed that his transition had led him to appreciate his male identity even more:

Because things that are obvious to cis people aren’t obvious to me; rather, I’ve had to figure out and struggle with all these apparent things for quite a few years […] So I feel like I can appreciate myself as a man more than I think I would have if I had been born male. (William, man, 20)

While William reflected on his ability to appreciate his male identity, another trans man, Edvin (28), stressed that settling into his male identity allowed him to be “more feminine than I ever was before.” A growing self-confidence made him comfortable enough to shed “tough and aggressive” masculinity. Finding an authentic self could also mean letting go of rumination about gender, as for Mika (non-categorized gender, 32) who settled into being “part of the Earth” in a “liberating self-dissolution.”

Many participants stressed that finding and becoming their authentic selves had led them to develop a huge amount of courage that, in turn, led them to “dare to explore more, not just in trans contexts, dare to be braver” (Samuel, man, 27). Several participants emphasized that a TGD experience by necessity meant that they had had to process things that cisgender people would not have to reflect on, but as Yoshi (nonbinary, 26) stated, “I think it’s […] so worth it because you learn so much about yourself.”

### Personal development

All participants stressed that a transition process and/or settling into a gender identity other than their assigned gender included a personal development that had been important for them. A number of new skills were mentioned by participants, from being “comfortable speaking to an audience [and] gaining leadership qualities” (Emil, man, 30), to being open with other minority experiences, as for Katja (woman, 35), who claimed that “It wasn’t a very big thing to come out as a lesbian […] [and] as polyamorous […] because if they can accept me as a trans person, they can somehow accept me as a lesbian, nonbinary, polyamorous, queer trans person.” Noa, in turn, described the courage gained from a new self-confidence:

[I] feel like I’ve developed… the courage to, you know, make demands, ask questions, stand up for myself in various ways, and so on. […] I can’t imagine what kind of person I would have been if I hadn’t had these experiences. […] it feels like I’d be, you know, not as strong a person, not as confident in myself. (Noa, genderfluid, 31)

While many saw courage as stemming from gained self-confidence, Kalle (man, 38) felt it came from a “humility toward life and understanding that life can take you on a journey you never imagined for yourself” where being trans had helped him to “dare […] to not just follow the well-trodden paths.”

Some participants highlighted personal growth through difficulties, such as William (man, 20), who described how proud he was that he had transitioned despite being “very shy, a bit introvert, very scared of everything more or less, social anxiety, needle phobia […] it’s cool to know that I did all this alone.” Likewise, Liam described his feeling of growth and pride:

I’ve gone through a transition, so what could be harder? Of course, there are many things that could be more difficult, but I still have the feeling of “Ah, how bad can it be? I’ll get through it.” (Liam, man, 51)

An aspect of personal development commonly mentioned among participants with binary identities was unique insights from the experiences of having been coded as both binary genders. Such bi-gendered experiences had given a “much broader base of experience” (Kalle, man, 38), because “if you have lived as two genders, you can see yourself as bilingual” (Liam, man, 51). Amanda (woman, 44) described her bi-gendered insights as “creating a deeper understanding of people in general.”

A specific form of bi-gendered insight was a deeper understanding of gender inequality, personally experienced by participants who previously had been coded as another gender. While insights about inequality were not positive in themselves, they led participants to develop norm-critical perspectives and open minds, which in turn was experienced as positive, leading to “a better understanding, more broad-mindedness, when it comes to people and the enormous variation of humanity” (Alex, genderqueer, 37). Lisa explained how her TGD identity had led her to develop a critical mind:

It has made me more critical in my thinking […]. I believe that on a purely moral level, when you’re forced to think more critically, you develop a more nuanced moral understanding and might become kinder toward others. So, I find a lot of joy in that. (Lisa, woman, 20).

Lisa described how her developed moral thinking had led her to be a better person. This is also the focus of the following subtheme.

### Contributing to a better society

Many participants felt that their TGD experiences gave them unique opportunities to contribute to a more open and friendly society, which was experienced as deeply meaningful. While some saw themselves as role models for other, younger, TGD people and helped organize meeting spaces, many extended their contributions beyond TGD related topics. Several participants said that being openly TGD often made others comfortable sharing their own minority experiences, since a TGD individual was seen as “a friend who is open-minded […] who won’t judge you” (Emil, man, 30), or as Adam (man, 25) explained, knows what it is like being “unfairly treated [and] forced into certain roles,” and therefore will be able to “put oneself in someone else’s shoes.” Several participants emphasized that their experiences fostered kindness and empathy, especially toward other minority people, as Hans explained:

It has given me greater empathy for other minorities as well. […] When I encounter issues related to other minorities that I don’t belong to myself, I have a strong tendency to want to listen, I want to learn, and to let them take up space with their experiences if they want to. […] And that perspective helps me in my life when I’m trying to be a better person toward my fellow human beings, so in that way, it has been very enriching. (Hans, man, 28)

Several participants highlighted that being role models for others was positive. Jenny saw the importance of being a role model for her students:

When I worked at [name of university], I felt it was actually important that I, as a visible part of the LGBTQ+ community, was actually teaching. It’s a living example of diversity, so to speak. And I think it’s very important for us as minorities to be visible and to expand the normality. (Jenny, woman, 54).

For Jenny, it had felt particularly meaningful when she realized that her LGBTQI+ students felt safe to come out in her class, “that I give the opportunity, simply through my presence, for others to open up.” Likewise, Alex (genderqueer, 37), said it was “rewarding to […] inspire people to be more true to themselves” when a younger person thanked them for inspiring them to come out as nonbinary.

While Alex pointed at the importance of role models for TGD people, Magnus (man, 45) stressed that TGD people may be a role model for anyone undergoing change in life, and “may inspire [people] to tune into what they truly want […] maybe change career or whatever it may be […] you inspire people to be more themselves.” In a broader sense, Emil (man, 30) stressed that being trans made him realize that homogeneity is “incredibly damaging, as the more similar we are, the narrower our access to [other] perspectives.” Even more so, Mika described TGD insights as liberation for all people:

Trans isn’t just about liberation for trans people; it’s about liberation for everyone, from a sort of violent norm. The cisnorm is, like, so violent in many ways. You don’t need to identify as trans to find what I’m saying liberating. (Mika, non-categorized gender, 32)

Mika depicted cisnormativity as violent to all humans, as it forces everyone into stereotypic gender roles. With TGD experiences, a less violent world could be envisioned.

## Minority peace

The theme minority peace focuses on how participants’ TGD identity or experiences lead to feelings of relaxation, comfort, and peace. The subtheme *Resting from mental ill health* depicts the calmness and peace felt after overcoming mental ill health. The subtheme *Relaxing in safe environments* describes peaceful and relaxing feelings from being in safe or affirmative environments, sometimes in contrast to a less validating or hostile surrounding. The subtheme *Personal stability* focuses on the peacefulness of feeling grounded as a TGD person.

### Resting from mental ill health

Many participants described periods of mental ill health, commonly related to struggling with their identity, and/or gender dysphoria. While these experiences were described as very severe, participants also emphasized the significant contrast once they settled into their identity, and/or received desired gender affirmative care. This led to a greater appreciation of life, as “the relief when dysphoria disappears or decreases, there comes a form of happiness from that relief” (Katja, woman, 35). For Pontus, settling into his male identity had been life changing:

I have one thing that tops everything else, and that is… before I understood that I was trans, I wanted to die. Every day I wanted to die. […] Since I realized that I am Pontus, I haven’t wanted to die, and I haven’t had any depression. (Pontus, man, 52)

As Pontus described, finally being free from depression was the one thing topping all positive experiences in life, and for him, settling into his TGD identity was the key to wellbeing. Another participant, Samuel, explained that while the absence of mental illness is life changing in itself, it also leaves energy for anything else that may bring joy or meaning to life:

For me personally, it’s one of the biggest aspects—of course, not having anxiety anymore, but also […] daring to explore all the other positive things that I now have the energy, time, and motivation for. And that leads to all sorts of things. It makes everything more enjoyable, and it leads to me daring to do more things. (Samuel, man, 27)

Being able to rest from burdensome experiences of mental ill health made Samuel able to focus his energy on all the meaningful and positive parts of life. Hence, in this theme, participants’ experiences of calm are generally set in contrast to earlier negative experiences, as a breathing space that would not be necessary if minority stressors were not so prevalent in TGD people’s lives.

### Relaxing in safe environments

Several participants described serene feelings experienced in safe and secure environments, where they were validated in their identity, and did not fear stigmatization. For many, the TGD community offered such a space, where advice, support, and practical help is never far away, as “I know safety is there whenever I need it” (Lisa, woman, 20). Joan also highlighted the importance of support from the TGD community:

If I write about something difficult that happened at work, I know I’ll get at least 10…20 comments from people, ranging from a trans person I met once at Pride and who has been following me since then, to my best friend. There’s this strong community support where I don’t need to know someone very well to receive compassion and support, and I find that there is a lot of that in the trans community. (Joan, nonbinary, 30)

Joan described the TGD community as a kind space, where they could find support whenever exposed to negative experiences. Likewise, several participants emphasized the importance of having safe spaces, because” at the end of the day, you just want to meet other trans people and sit together, feeling natural in a way, without having to explain or educate” (Michele, agender, 22). Michele went on explaining why the TGD community offered so much relaxation for them:

I’ve started to hang out in this gender-fuckery community, which doesn’t cater to a cis audience. And it’s completely okay for people to cater to a cis audience […] But there’s more to it. (Michele, agender, 22)

Within the “gender-fuckery” community, Michele had found a space where they could interact on their own terms, not considering what any cisgender person would think of them.

Some participants mentioned other spaces than the TGD community where they felt relaxed or validated and where they could rest from stressors, such as among family, friends, or colleagues, but also among strangers in environments that felt calm and safe. Anders found nude beaches to be a relaxing space:

I’ve started going to nude beaches in recent years. And it’s also because I feel so comfortable there; it’s incredibly relaxing. So I’ve almost sought out environments where I can feel especially at ease in my body. (Anders, man, 42)

It is worth pointing out that Anders found relaxation in a space where the body is fully uncovered, i.e. where any bodily aspects not in line with normative expectations would be visible. Still, he stressed that this is where he can feel “especially at ease in my body”, showing the importance of places where bodies of different forms and shapes are welcomed in a nonjudgemental context.

In contrast to those who, like Anders and Michele, highlighted specific arenas where they felt calm and relaxed, one participant, David (man, 45), described a similar feeling of being validated and safe in Swedish society in general which “can be very open and even participatory in a way and like genuine.” This gave him “a renewed faith in humanity.” David had recently come out as a transgender man and was surprised that his transition had been so well received.

In addition to the importance of being able to relax in the company of other people, one participant, Mika (non-categorized, 32), described how they felt at peace in contact with non-human animals and plants, as in these relations you are “not gendered, not read or categorized in that sense.”

### Personal stability

Peacefulness was in many interviews described in relation to a personal stability gained from settling into a TGD identity. As described in the theme *Minority growth*, under the subtheme *Being authentic*, many participants described how courage and self-confidence had been developed as a result of personal growth. The present theme emphasizes how the improved self-confidence led to an inner peacefulness, where *“*I listen to myself more*”* (Kalle, man, 38). Samuel described how gained self-confidence led to an inner stability and safety:

When you already have a label on you, you’re not afraid of being labeled more, because then it just turns into tattoos, and you can turn it into something [positive]. I mean, that sense of freedom is really what it is […] society doesn’t scare me as much, or norms don’t scare me as much. (Samuel, man, 27)

For Samuel, breaking norms had become less frightening since he already violated cis norms, which gave a sense of freedom. While this is close to the metaphor of having access to a “buffet” of gender expressions, here there is a clear description of processing societal norms and freeing oneself from them. Another participant, Kalle, explained how his TGD experiences had brought him closer to his own emotions:

I think I’m much closer to my emotions than I would have been otherwise, because I wouldn’t have dared to be otherwise. That’s something I reflect on and am grateful for. (Kalle, man, 38)

While many participants stressed that gender affirming care had been important in their process of reaching personal stability and thriving in life, a few participants had realized that their bodily dysphoria declined once they found inner peace in their gender identity, as “when I look in the mirror […] I see myself as a man. […] objectively, I see that I look like a woman, but behind that I nevertheless see the man” (David, man, 45).

Some participants with a binary gender identity described how they had become more comfortable in their identities over time and expressed a sense of peace in relation to this. Selma had transitioned in her late teens and early twenties. Over the years since, she had concealed her transgender background from new social contacts. Lately she had, however, started to disclose her background to friends and colleagues. She said:

This kind of second wave of coming out […] that I’ve experienced since last summer, is a pretty big deal for me and my life. This self-acceptance, becoming more okay with being trans and being a trans woman, and being able to talk openly about it—it’s like a huge thing. (Selma, woman, 29)

Inspired by other transgender women, Selma had begun to disclose her transgender background, a decision that had improved her self-acceptance. Her narrative shows how a stepwise settling into a TGD identity can bring a sense of minority peace, or, as Samuel (man, 27) put it *“*[over time] it’s less of a gender euphoria, and more of a trans euphoria*.”* Thus, rather than feeling strongly about being a man, Samuel felt an increased pride in being TGD.

## Discussion

In the present study we invited TGD people to reflect on and describe positive experiences connected to being TGD. The in-depth interviews allowed for nuanced exploration, and the large interest and positive feedback we received from participants confirmed to us that this is an important area of knowledge production.

The analysis resulted in three main themes of which one was in line with the concept of minority joy, while the other two were better described as a sense of minority growth and minority peace. Content wise, results show a diverse range of positive experiences associated with being TGD, consistent with earlier studies (Flynn et al., 2024; Riggle et al., 2011; Shuster & Westbrook, 2024). Results suggest that the three themes are distinct from each other, even if they are likely to interact and may be experienced simultaneously. While it is conceivable that experiences of minority joy, growth, or peace may be connected to the ability to cope with a stressful situation or to greater resilience, they can also be understood as theoretically distinct from these concepts. For example, both coping and resilience are usually connected to negative experiences, while our results include positive experiences not directly related to stressors. The focus on emotional valence and the resulting differentiation of positive experiences can inform this area of research going forward.

The emotional valence of experiences is an important aspect to differentiate the themes in our result. To further theorize the differences between minority joy, minority growth, and minority peace, we have turned to positive psychology. Many positive psychologists (e.g. Fredrickson, 2001; Huta & Ryan, 2010; Huta & Waterman, 2014) base their understanding of wellbeing and life satisfaction on Aristotle’s classic division between eudamonia and hedonia (Aristotle, 2009). Minority joy aligns with hedonia, focusing on pleasurable experiences, positive feelings, and moments where life is savored. Participants described this when they enjoyed community, experienced gender euphoria, and felt a sense of freedom and playfulness that they had access to because of being TGD.

Minority growth, on the other hand, aligns with eudamonia, which includes a movement toward “growth, authenticity, meaning and excellence” (Huta & Waterman, 2014, p. 1448). It relates to the term stress related growth, which is understood as the changes experienced as a result of stressors (Szymanski et al., 2023). Participants described this growth as achieving authenticity, both in the sense of settling into an authentic identity, and in the sense of living authentically, including social relationships. This is in line with how Riggle and Mohr (2015) understand authenticity. Participants also described a wider personal development, feeling brave and proud of their achievements. Many described that the personal growth included a moral development, and that this gave them the opportunity to widen their horizons and contribute to a better society. In conclusion, minority growth involves reflexivity and personal stability, enabling participants to live on their own terms rather than being dependent on society’s norms and expectations.

The theme minority peace, characterized by tranquility and being at rest, is qualitatively different from the happy and joyful feelings of minority joy, and from the aspects of authenticity and development covered by minority growth. While minority peace aligns with eudaimonia, in the sense of living a good life, it was typically described as “just being” without having to do or think, similar to tranquility (Ellsworth & Smith, 1988), contentment (Fredrickson, 1998) or ataraxia (Striker & The Hegeler Institute, 1990).

Some subthemes described positive experiences outlined in contrast to negative ones, such as growth from difficult experiences or relaxation in safe spaces. In line with previous studies (Beischel et al., 2022), gender euphoria was described in contrast to, but also as being present simultaneously with gender dysphoria. Several participants described the euphoric feeling of being socially affirmed in their gender identity, which previous studies have connected to positive health outcomes (King & Gamarel, 2021; Olson et al., 2016). Generally, TGD people experience a disproportionate amount of social stress due to the transphobia and cisnormativity prevalent in society (Linander et al., 2024; Lundberg et al., 2023). This also highlights the importance of taking the whole picture into account. For example, while some of the trans women in our study described having been raised as male as a privilege, it is important to stress that gender is constructed socially and subject to changes both contextually and over time. These gendered structures tend to have a particularly negative effect on trans women’s lives in the form of potentially high rates of violence (Westbrook, 2022). In summary, positive and negative experiences sometimes coexist and influence each other: without minority stress, safe spaces would not be necessary, and the personal development our participants described would not have taken this specific course.

In contrast, many experiences were described as positive in their own right, and possible to access regardless of personal experiences of minority stress. For example, having access to community was described as a joyful and enriching benefit of being TGD *and* as a place for support and relaxation, where participants could rest from stressors. The fact that most participants mentioned TGD community as a source of positive emotions highlights the community’s role in buffering against ill health, which is in line with previous studies (Pflum et al., 2015; Sherman et al., 2020).

Participants’ narratives highlight the importance of not expecting all TGD people to have the same experiences. For example, participants having received gender affirming care clearly described how this had a huge positive impact on them and connected it to gender euphoria and personal development. This is in line with earlier research highlighting the positive effects of gender affirming treatment for TGD youth and adults (Almazan & Keuroghlian, 2021; Tordoff et al., 2022) and underlines the importance of making care available to TGD people. At the same time, some participants did not feel the need for gender affirming care after reaching a place where they felt more grounded in their identity. Also, some narratives included positive experiences in arenas that previously have been described as particularly stressful for many TGD people, such as visiting nude beaches or choosing public restrooms (Caudwell, 2022; Lewkowitz & Gilliland, 2025). Such variety deconstructs the homogeneity central to available discourses regarding TGD issues.

In addition to positive psychology, themes related to minority joy, growth, and peace can also be understood through clinical frameworks such as Gilbert’s (2009) theory of systems of regulation of affection (threat, drive, and soothing) used in compassion focused therapy (CFT). Minority stressors typically activate the threat system, but also—depending on the emotion evoked—the drive system to enable resistance. Minority joy and minority growth invoke similar aspects connected to the drive system, while minority peace involves the soothing system. Building on CFT-theory and on tools used in clinical settings, integrating these insights into clinical sessions can enhance TGD-informed and person-centered approaches to address client’s unique experiences with these different systems.

In our study, positive experiences were not only described as buffers against ill health (even though this aspect was also present) but as positive in themselves and as important for quality of life. This is an important addition to earlier clinical and research models explaining the development of ill health in TGD/LGBTQI+ people, where positive aspects generally are understood as protective factors acting as mediators between stressors and health outcomes (Meyer, 2003; Testa et al., 2015). Recognizing TGD people’s diverse positive experiences is essential to counteract the trope of the transgender person in misery (Shuster & Westbrook, 2024) prevalent in media, health care and (sometimes) in TGD people’s own perception (Corbett, 2023). This could have a profound influence on professional development, where competency is sorely needed, and could improve the experience of TGD people when meeting professionals in different capacities.

### Study limitations

The study has limitations that need to be considered in relation to the transferability of the results. For example, while both nonbinary and binary identities were included, more trans men than women participated and all but one of the nonbinary participants were assigned female at birth. Also, only three participants reported belonging to an ethnic minority, all but three were born in Sweden and most had a university education. As such, discerning patterns in sub-groups of participants was not possible. Nevertheless, these results can serve as inspiration for further quantitative studies that can include more diverse groups of TGD people and explore the prevalence of different kinds of positive experiences in different subgroups as well as potential associations with health outcomes.

### Practical implications and recommendations

We strongly recommend that health care practitioners and social workers routinely ask questions about the diverse positive experiences of their TGD clients and make use of these strengths when planning further interventions. As described in previous studies, gender euphoria and community connectedness were experienced as beneficial for health and quality of life (Beischel et al., 2022; Pflum et al., 2015; Sherman et al., 2020). For example, our participants described how gender euphoria, in line with Beischel et al. (2022), was experienced connected to physical, psychological, or social aspects, which practitioners can make use of by using the correct language and going out of their way to affirm gender where possible. For researchers, much remains to be explored regarding TGD people’s positive experiences. For example, studies with larger sample sizes would be beneficial studying different TGD populations further, while also focusing on different sociocultural contexts, class, experiences of racism, functional impairments, and other important intersections. Also, since the area of positive experiences related to TGD people’s minority identities is still theoretically underdeveloped, additional work is needed to highlight the diversity of positive experiences and to enable the development of clinically useful models and interventions to further improve TGD people’s health and quality of life. In summary, the results of our and similar studies raise awareness of the multifaceted positive experiences and potential for personal development available to TGD people. Not the least, this is important for policy makers who need to be made aware of these results that are, as yet, not part of general knowledge in society, but which contain important information for future reforms.

## Conclusions

- TGD people have a variety of positive experiences connected to being TGD.
- These experiences have different emotional valences and can be understood as minority joy, minority growth, and minority peace.
- Some of these experiences are connected to stressors, but some are better understood as advantages of being TGD.
- An awareness of these positive experiences can inform professionals, TGD people themselves, and policy makers.
